# Whey Protein Isolate-Supplemented Beverage, Fermented by *Lactobacillus casei* BL23 and *Propionibacterium freudenreichii* 138, in the Prevention of Mucositis in Mice

**DOI:** 10.3389/fmicb.2018.02035

**Published:** 2018-09-12

**Authors:** Bárbara F. Cordeiro, Emiliano R. Oliveira, Sara H. da Silva, Bruna M. Savassi, Leonardo B. Acurcio, Luisa Lemos, Juliana de L. Alves, Helder Carvalho Assis, Angélica T. Vieira, Ana M. C. Faria, Enio Ferreira, Yves Le Loir, Gwénaël Jan, Luiz R. Goulart, Vasco Azevedo, Rodrigo D. de O. Carvalho, Fillipe L. R. do Carmo

**Affiliations:** ^1^Institute of Biological Sciences, Federal University of Minas Gerais (ICB/UFMG), Belo Horizonte, Brazil; ^2^STLO, INRA, Agrocampus Ouest, Rennes, France; ^3^Institute of Biotechnology, Federal University of Uberlândia, Uberlândia, Brazil

**Keywords:** *Lactobacillus*, mucositis, probiotics, *Propionibacterium*, stress tolerance, whey protein isolate

## Abstract

Mucositis is a clinically important gastrointestinal inflammatory infirmity, generated by antineoplastic drugs cytotoxic effects. The inflammatory process caused by this disease frequently leads to derangements in the alimentary tract and great malaise for the patient. Novel strategies are necessary for its prevention or treatment, as currently available treatments of mucositis have several limitations in relieving its symptoms. In this context, several research groups have investigated the use of probiotic bacteria, and in particular dairy bacterial strains. Compelling evidences reveal that milk fermented by certain probiotic bacteria has the capacity to ameliorate intestinal inflammatory disorders. In addition, innovative probiotic delivery strategies, based on probiotics incorporation into protective matrices, such as whey proteins, were able to increase the therapeutic effect of probiotic strains by providing extra protection for bacteria against environmental stresses. Therefore, in this study, we evaluated the role of the whey protein isolate (WPI), when added to skim milk fermented by *Lactobacillus casei* BL23 (*L. casei* BL23) or by *Propionibacterium freudenreichii* CIRM-BIA138 (*P. freudenreichii* 138), as a protective matrix against *in vitro* stress challenges. In addition, we investigated the therapeutic effect of these fermented beverages in a murine model of mucositis induced by 5-Fluorouracil (5-FU). Our results demonstrated that milk supplementation with 30% (w/v) of WPI increases the survival rate of both strains when challenged with acid, bile salts, high temperature and cold storage stresses, compared to fermented skim milk without the addition of WPI. Moreover, treatment with the probiotic beverages prevented weight loss and intestinal damages in mice receiving 5-FU. We conclude that the presence of WPI maximizes the anti-inflammatory effects of *L. casei* BL23, but not for *P. freudenreichii* 138, suggesting that whey protein enhancement of probiotic activity might be strain-dependent.

## Introduction

Mucositis is a severe inflammation that affects the entire extension of the Alimentary Tract (AT) of individuals undergoing malignancy treatment based on chemotherapy or radiotherapy (Sonis, 2004). One of the main drugs associated with this condition is 5-Fluorouracil (5-FU). This is a antimetabolic drug commonly prescribed for the treatment of head, neck and gastrointestinal cancer (Longley et al., 2003). 5-FU unfortunately presents non-specific cytotoxicity toward cells, inhibiting the proliferation of both cancer cells and normal cells with high replication rates, such as the enterocytes of the gastrointestinal tract (GIT) (Carvalho et al., 2017a). A series of clinical symptoms, such as nausea, weight loss, vomiting, severe abdominal pain and diarrhea are commonly reported in patients receiving 5-FU during cancer treatment (Bastos et al., 2016). Moreover, mucositis frequently increases predisposition to local and systemic secondary infections, thus generating additional costs and extending the patient’s hospitalization time (Carvalho et al., 2017a). Mucositis is characterized by pathological changes in the small bowel. These changes include the presence of degenerate enterocytes (Ciorba et al., 2016), submucosal vessel damage, leukocyte infiltrate in the *lamina propria*, with accumulation of neutrophils and eosinophils (Antunes et al., 2016), increased mucin production and degeneration of goblet cells (Stringer, 2013), atrophy of villi, hypoplasia and apoptosis of intestinal crypts (Chang et al., 2012). Currently, there is no treatment that is completely successful in the prevention and treatment of mucositis. However, there has been a growing interest in the use of probiotics as promising candidates for the treatment of this disease (Carvalho et al., 2017a).

Probiotics are included in a variety of products, including fermented foods, dietary supplements, formulas for newborns and infants, as well as various pharmaceutical formulations (Cousin et al., 2011). Currently, fermented beverages by one or more bacteria, have gained the functional food status, which makes them an important part of our diet as well as our main daily source of beneficial microbes (Leroy and De Vuyst, 2014; Carmo et al., 2017).

Selected strains of lactic acid bacteria (LAB) were reported as probiotic with beneficial effects provided by different mechanisms of action and can be used in functional foods withal (Carvalho et al., 2017b; Eales et al., 2017; Tang et al., 2017). Some studies have shown that the administration of lactobacilli strains can reduced some parameters of mucositis in mice model induced by 5-FU, such as prevent weight loss, attenuate the diarrhea and intestinal damage (Justino et al., 2015). *L. casei* BL23 has also been considered as a good probiotic strain, according to results obtained in others inflammatory models. Some studies demonstrated that *L. casei* BL23 was able to alleviates colitis symptoms in a dextran sulfate sodium (DSS) model (Foligne et al., 2007; Rochat et al., 2007) and the ability of these strain to attenuate intestinal inflammation can be enhanced using a protection matrix (Lee et al., 2015b). Other important group of bacteria widely used in the food industry, particularly in Swiss-type cheese manufacture, is propionic acid bacteria (PAB). *Propionibacterium freudenreichii* represents the main species thereof and is listed in the Qualified Presumption of Safety list (QPS) by the European food safety authority and it has recently been considered a promising probiotic (Rabah et al., 2017). *P. freudenreichii* produces metabolites are considered as prebiotics, such as 1,4-dihydroxy-2-naphtoic acid (DHNA) and 2-amino-3-carboxy-1,4-naphthoquinone (ACNQ), both associated to bifidogenic effects. Furthermore, *P. freudenreichii* is the only GRAS bacterial species producing food-grade vitamin B12 at the industrial scale (Rabah et al., 2017). Selected *P. freudenreichii* strains have been associated with therapeutical effects based on *in vitro* and *in vivo* properties to attenuate colitis model induced by trinitrobenzene sulfonic acid (TNBS) (Cousin et al., 2012a, 2016; Plé et al., 2015). A dairy propionibacteria, *P. freudenreichii* 138, demonstrated a pro-apoptosis capacity, in HTG-1 human gastric cancer cells without toxicity effects in healthy human cells (Cousin et al., 2012a, 2016). Moreover, Cousin et al. (2012b) shown the persistence of *P. freudenreichii* 138 in piglets colon, withal metabolic activity and producing propionate, which is a SCFA with probiotic properties (Cousin et al., 2012b).

An extremely important factor for the therapeutic effects of probiotics is the ability of the bacteria to survive during transit through the GIT or during industrial processes (Cousin et al., 2012b; Rabah et al., 2017). Digestion indeed imposes harsh conditions including gastric acid and presence of bile salts, which may severely affect bacterial viability (Leroy and De Vuyst, 2014; Huang et al., 2016a). A probiotic microorganism must, however, tolerate these stresses for a long persistence in the host and for an enhanced beneficial effect (Carmo et al., 2017). In the aim to maximize the tolerance of bacteria to stressful environments and thus to increase their probiotic ability, the food matrix used in the manufacture of fermented products plays a key role as a protective medium (Gagnaire et al., 2015; Lee et al., 2015a). As an example, milk proteins, as well as whey protein isolates, constitute very promising protective matrices for probiotics, besides being an efficient delivery vehicles to target protective molecules and microorganisms to digestive epithelium (Livney, 2010; Cousin et al., 2012a; Vargas et al., 2015; Huang et al., 2016a). Whey proteins have been recognized for their various functional and nutritional properties. The functional properties are mainly due to their physical, chemical and structural characteristics and the nutritional value is directly linked to the concentration of essential amino acids (Yadav et al., 2015). Some studies have also demonstrated the potential of whey proteins to enhance the survival and viability of probiotic bacteria during production and storage (Marshall, 2004; Madureira et al., 2007; Almeida et al., 2009; Huang et al., 2016b; Baruzzi et al., 2017; Dąbrowska et al., 2017).

The aims of this work were (i) to evaluate whether whey protein isolate is a good protective matrix for *L. casei* BL23 and *P. freudenreichii* CIRM-BIA 138, against adverse environmental conditions and (ii) to investigate the therapeutic effect of administration of whey protein isolate-supplemented beverage, fermented by both strains, in the prevention of mucositis induced by 5-FU.

## Materials and Methods

### Bacterial Strains and Growth Conditions

*Lactobacillus casei* BL23 strain was kindly supplied by the UMR1219 Micalis Institute (INRA-AgroParisTech, Jouy-En-Josas, France) and the *P. freudenreichii* CIRM-BIA138 (alias ITG P9) strain by the Biological Resource Center (International Center of Microbial Resources, INRA, Rennes, France). *L. casei* BL23 was grown in MRS broth at 37°C for 24 h, without shaking. *P. freudenreichii* 138 was grown in YEL culture medium at 30°C for 72 h, without agitation (Malik et al., 1968).

### Dairy Beverage Formulation and Supplementation With Whey Protein Isolate

The fermented beverage was prepared using skimmed milk (SM) powder 12% w/v (Itambé, Brazil). For cultivation of *L. casei* BL23 (SMLC), the SM was supplemented with yeast extract (Kasvi Curitiba, Brazil) and glucose (Merck, Germany) (Tharmaraj and Shah, 2003). For cultivation of *P. freudenreichii* 138 (SMPF), the milk was supplemented with casein peptone (5g/L) (KASVI, Curitiba, Brazil) and sodium lactate (50 m/M) (Sigma, St. Louis, MO, United States) (Cousin et al., 2012b). Both milk were autoclaved at 110°C for 15 min. The SMs was supplemented with whey protein isolate (WPI), natural flavor, 90% protein (Vulgo Supplements, Brazil) at concentrations of 5, 15, and 30% w/v, and strains cultivated in skimmed milk without WPI was used as control. The same growth conditions used to cultivate the strains in MRS or YEL were applied for growth in fermented beverages.

### Stress Challenges

Samples of 10 ml from the stationary-phase of *L. casei* BL23 and *P. freudenreichii* 138 in culture media or in skim milk, supplemented or not with WPI were subjected to acid, bile salts, and heat challenges (Huang et al., 2016b). For acid stress, the samples were incubated in MRS broth or YEL broth, previously adjusted to pH 2.0 using HCl, at 37°C for 60 min. Briefly, for bile salts stress, the samples were incubated in MRS broth or YEL broth containing 1.0 g/L of bile salts (an equimolar mixture of cholate and deoxycholate, Sigma Chemical, St. Louis, MO, United States) and then, incubated at 37°C for 60 min. Finally, to simulate the pasteurization temperature established by the International Dairy Foods Association (IDFA), the samples were incubated in their specific culture media pre-heated to 63°C for 30 min. After stresses challenges, aliquots of each sample were subjected to 1:10 serial dilutions using peptone water (9 g/L peptone, 5 g/L NaCl) and plated on MRS agar or YEL agar medium. Plates of *L. casei* BL23 were incubated for 48 h at 37°C. Plates of *P. freudenreichii* 138 were incubated for 144 h (6 days) at 30°C in jars containing anaerobiosis generator (Anaerocult A^®^, Merck Millipore). The number of viable bacteria was determined by counting of colony forming unit (CFU) after incubation. The bacteria survival rate (%) through each stress condition was calculated through the following equation (Ferreira et al., 2017):

$$\text{Survival Rate (\%) : }\frac{\log\;N}{\left(\log\;N_{0}\times 100\right)}$$

Where *N* refers to the number of bacteria population (CFU mL^-1^) in culture medium after stress challenges, and *N*_0_ refers to the number of initial population (CFU mL^-1^) before the stress challenges.

### Bacterial Survival During Storage at 4°C

The long-term survival of *L. casei* BL23 and *P. freudenreichii* 138 in dairy beverage supplemented with 30% of WPI was assessed during the storage process at 4°C for 90 days kept away from light (Huang et al., 2016b). For the evaluation of bacterial survival during cold storage, plate seeding was performed on days 0 (pre-storage time), 7, 14, 21, 30, 60, and 90 after storage. The *L. casei* BL23 and *P. freudenreichii* 138 plates were incubated according to their specific conditions on agar media (see above). The number of viable bacteria during storage was determined by counting CFU in the culture after incubation. The evolution of acidification of stored samples was also screened in the same days. To evaluate if both fermented beverages could survive GIT stress conditions after being stored at 4°C, we performed acid stress and biliary stress *in vitro* challenges for the dairy beverage supplemented with 30% of WPI. Acid and bile salts stresses were performed on days 30, 60, and 90 after the storage start.

### Evaluation of Therapeutic Effects of Beverages Fermented by *L. casei* BL23 or *P. freudenreichii* 138 in a Mice Model of Mucositis

#### Animals

Conventional female BALB/c mice between 6 and 8 weeks of age were obtained at Federal University of Minas Gerais (UFMG–Belo Horizonte, Brazil). Mice were kept in a temperature-controlled room with *ad libitum* access to water and standard chow diet. The study was approved by the Ethics Committee on Animal Experimentation of the Federal University of Minas Gerais (CEUA-UFMG, Brazil, protocol 366).

### Probiotic Treatment, Mucositis Induction, and Experimental Groups

For probiotic treatment, mice received 0.5 ml of fermented beverages supplemented or not with 30% of WPI via gastric gavage, during 13 days. In order to induce mucositis, mice received a single intraperitoneal injection of 5-FU (Fauldfluor – Libbs) (300 mg/kg) on day 11, and were euthanized 72 h after induction of mucositis, in 14th of experimental day (Carvalho et al., 2017a). An injection of saline (NaCl 0.9%) was used in control groups. After euthanasia, a longitudinal abdominal incision was performed to remove the intestine for further analyses. Body weight of mice was determined throughout the experiment. For *in vivo* experimentation, BALB/c mice were divided into sixteen groups according to **Table 1**. All experiments were performed simultaneously therefore, the same control groups were used for all experimental probiotic assays. Each group containing 6–9 animals. All beverages contained 10^9^ CFU mL^-1^ bacteria.

**Table 1:** Experimental groups and the respective treatments.

Non-inflamed groups Injection of 300 mg/kg of saline (0.9% NaCl)		Inflamed groups Injection of 300 mg/kg of 5-FU	
Group	Treatment	Group	Treatment
Water	H_2_O	Water	H_2_O
SMLC + WPI	Skim milk specific for *L. casei* BL23 + WPI	SMLC + WPI	Skim milk specific for *L. casei* BL23 + WPI
SMPF + WPI	Skim milk specific for *P. freudenreichii* 138 + WPI	SMPF + WPI	Skim milk specific for *P. freudenreichii* 138 + WPI
SMLC + BL23	Skim milk specific for *L. casei* BL23 fermented by *L. casei* BL23	SMLC + BL23	Skim milk specific for *L. casei* BL23 fermented by *L. casei* BL23
SMLC + WPI + BL23	Skim milk specific for *L. casei* BL23 fermented by *L. casei* BL23 + WPI	SMLC + WPI + BL23	Skim milk specific for *L. casei* BL23 fermented by *L. casei* BL23 + WPI
SMPF + 138	Skim milk specific for *P. freudenreichii* 138 fermented by *P. freudenreichii* 138	SMPF + 138	Skim milk specific for *P. freudenreichii* 138 fermented by *P. freudenreichii* 138
SMPF + WPI + 138	Skim milk specific for *P. freudenreichii* 138 fermented by *P. freudenreichii* 138 + WPI	SMPF + WPI + 138	Skim milk specific for *P. freudenreichii* 138 fermented by *P. freudenreichii* 138 + WPI
Association (Assoc)	Equal mixture of SMLC + WPI + BL23 and SMPF + WPI + 138	Association (Assoc)	Equal mixture of SMLC + WPI + BL23 and SMPF + WPI + 138

*All groups were gavaged daily, with 0.5 ml of the appropriate treatments, for 13 days.*

### Histological Analysis

The distal portion of the small bowel (ileum) from the mice was collected and washed with PBS. Afterwards, rolls were prepared for histomorphological analysis. Histological materials were immersed in 4% buffered formaldehyde solution for tissue fixation. Then, the material was embedded in paraffin, and a 4 μm section of each sample was placed on a glass slide and stained with Hematoxylin-Eosin (HE). The histological score was determined using a score that measures the intensity of the infiltrate of mononuclear and polymorphonuclear cells in the *lamina propria* of the ileum, the presence of ulceration and erosion and changes in mucosal architecture (Soares et al., 2008). For each parameter a classification was given according to the severity of the lesion in the tissues: absent (0), mild (1), moderate (2) and severe (3). For morphometric analysis, 10 images of the ileum of each animal were randomly captured and analyzed using ImageJ software (version 1.8.0). Villi height and the crypt depth were measured vertically from the tip of villi to the base of the adjacent crypt. Additional cuts in the paraffinized samples from the ileum were stained by the Periodic Acid-Schiff (PAS), technique to determine the number of goblet cells in the tissues (Prisciandaro et al., 2011). Ten random field images of each sample were made using the 40× objective and the intact goblet cells were counted using ImageJ software (version 1.8.0) and expressed as the number of cells per high-power field (hpf) (40×, 108.2 μm^2^).

### Measurement of Secretory IgA

Levels of secretory IgA (sIgA) were determined by enzyme-linked immunosorbent assay (ELISA) in small bowel intestinal fluids (Carvalho et al., 2017a). Microtiter plates (Nunc-Immuno Plates, MaxiSorp) were coated with anti-IgA antibodies (Southern Biotechnology, Birmingham, AL, United States) for 18 h at 4°C. The plates were washed with saline (NaCl 0.9%) added with Tween 20 (0.05%) and blocked with 200 μl PBS-casein (0.05%) for 1 h at room temperature. Intestinal fluid samples were diluted in PBS-casein (0.25%) and then added to the plate. After incubation for 1 h at room temperature, the wells were washed and biotin-conjugated anti-mouse IgA antibody (Southern Biotechnology) diluted in PBS-casein (0.25%) (1: 10,000). The plates were incubated for 1 h at 37°C and anti-IgA conjugated to streptavidin peroxidases (1:10,000) were added (Southern Biotechnology). After 1 h of incubation, 100 μl of orthophenylenediamine (OPD) (Sigma, St. Louis, MO, United States) and H_2_O_2_ (0.04%) were added to each well. Plates were kept away from light until the coloration developed. The reaction was stopped by addition of 2 N H_2_SO_4_. Reading was performed on a plate reader (Bio-Rad Model 450 Microplate Reader) at 492 nm absorbance. The results were measured in concentration of sIgA (μg) per ml of intestinal fluid, according to the standard curve.

### Statistical Analyses

The results were reported as the mean ± standard deviation and analyzed using Student’s *t*-test, Holm–Sidak *t*-test, One-Way ANOVA or Two-Way ANOVA followed by the Tukey or Sidak post-test. Non-parametric data’s were analyzed using Kruskal-Wallis data followed by the Dunns post-test. Graphs and statistical analyzes were performed in GraphPad Prism version 7.00 for Windows (GraphPad Software, San Diego, CA, United States). *P*-values under 0.05 were considered significant.

## Results

### Different Concentrations of Whey Protein Isolate Alter the Growth of Bacteria

**FIGURE 1:**
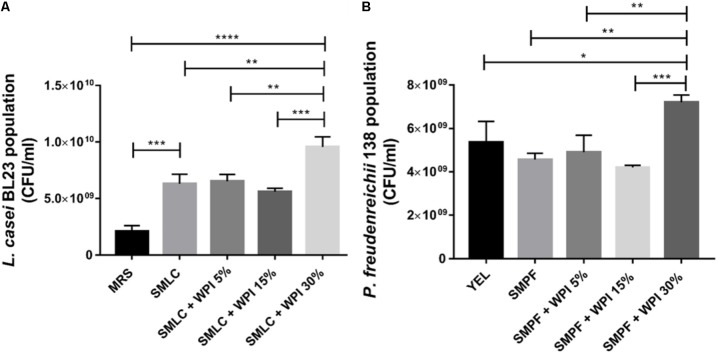
Different concentrations of WPI in skim milk impacted on *Lactobacillus casei* BL23 and *Propionibacterium freudenreichii* 138 growth. **(A)** Colony-forming unit (CFU) counting of *L. casei* BL23, after 24 h of incubation at 37°C and **(B)** Population of *P. freudenreichii* 138, after 78 h of anaerobic incubation at 30°C were assessed by CFU counts. These assays were performed in triplicate technique and biological triplicate. Asterisks represent statistically significant differences and were indicated as follows: ^∗^*p* < 0.05, ^∗∗^*p* < 0.01, ^∗∗∗^*p* < 0.001, ^∗∗∗∗^*p* < 0.0001.

The final population of *L. casei* BL23 and of *P. freudenreichii* 138 was monitored after growth in skim milk supplemented with different concentrations of WPI (**Figure 1**). We observed a significant increase in the CFU counting of *L. casei* BL23 when cultivated in SMLC after 24 h (6.3 × 10^9^ CFU mL^-1^), in comparison with MRS medium (2.1 × 10^9^ CFU mL^-1^). Moreover, the largest final population of *L. casei* BL23 was found in skim milk supplemented with 30% WPI (SMLC + WPI 30%) (9.5 × 10^9^ CFU mL^-1^). The final population of *P. freudenreichii* 138 also showed a similar result (**Figure 1B**). In this case, the highest population (7.2 × 10^9^ CFU mL^-1^) was obtained in skim milk supplemented with 30% of WPI (SMPF + WPI 30%). This condition allowed the highest growth of the propionibacteria, when compared with all other culture media used.

### Whey Protein Isolate Improves the Tolerance of Bacteria Toward Environmental Stresses

**FIGURE 2:**
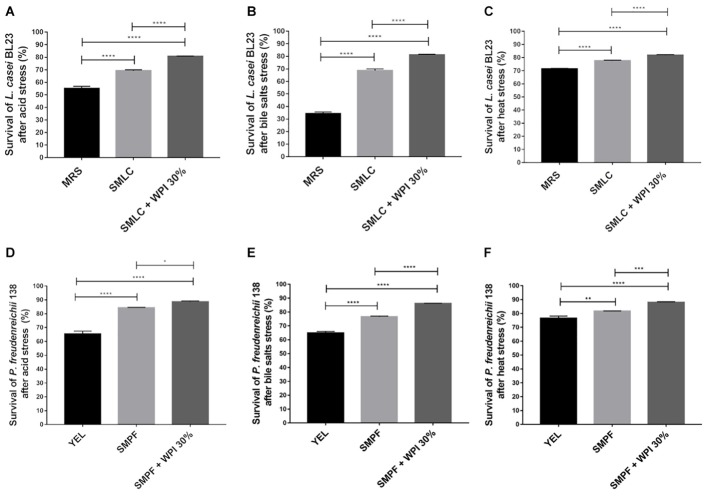
Whey protein isolate (WPI) confers stress tolerance on *L. casei BL23* and *P. freudenreichii* 138. *L. casei* BL23 was cultured for 24 h in the indicated growth media until stationary phase and then subjected to **(A)** acid stress (pH 2 for 60 min at 37°C); **(B)** bile salts stress (1 g/liter for 60 min at 37°C) or **(C)** heat stress (63°C for 30 min). *P. freudenreichii* 138 was cultured for 72 h in each culture media until stationary phase, and then subjected to **(D)** acid stress, **(E)** bile salts stress, or **(F)** heat stress. Viable bacteria were enumerated by counting colonies in the challenged and control cultures and then, expressed as percent survival (means ± standard deviations). These assays were performed in triplicate technique and biological triplicate. Asterisks represent statistically significant differences and were indicated as follows: ^∗^*p* < 0.05, ^∗∗^*p* < 0.01, ^∗∗∗^*p* < 0.001, ^∗∗∗∗^*p* < 0.0001.

The survival rate of *L. casei* BL23 and *P. freudenreichii* 138 was evaluated after acid stress, bile salts stress and high-temperature stress (**Figure 2**). *L. casei* BL23 showed enhanced survival rate after acid stress, when cultured in SMLC medium (69.2%), compared to the MRS control (55.1%) (**Figure 2A**). The survival was further increased when SMLC was supplemented with 30% WPI, leading to the highest survival rate of *L. casei* BL23 (80.6%). This value was significantly higher than skim milk supplemented with 5% and 15% of WPI (data not shown). The population after acid stress in SMLC + WPI 30% was 8.5 × 10^7^ CFU mL^-1^. A similar result was observed for *P. freudenreichii* 138 (**Figure 2D**). After acid stress, the highest tolerance was observed when *P. freudenreichii* 138 was grown in skim milk supplemented with 30% WPI (SMPF + WPI 30%) (88.6%), compared with skim milk without supplementation (SMPF) (84.3%) or YEL medium (65.3%). In addition, the survival rate of *P. freudenreichii* 138 grown in milk added with 5% of WPI or 15% are lower than supplementation with 30% (data not shown). The population of *P. freudenreichii* 138 after acid stress in SMPF + WPI 30% was 7.6 × 10^8^ CFU mL^-1^. Likewise, our results show enhanced tolerance toward bile salts stress when *L. casei* BL23 and *P. freudenreichii* 138 were cultured in beverage containing 30% of WPI (**Figures 2B,E**). After temperature stress, we observed that both strains presented a high tolerance to 63°C, independent of culture medium. However, the highest survival rate was obtained when bacteria were cultured in milk added with 30% of WPI (**Figures 2C,F**).

### Bacteria Remained Viable in the Fermented Milk Supplemented With WPI During Storage at 4°C

**FIGURE 3:**
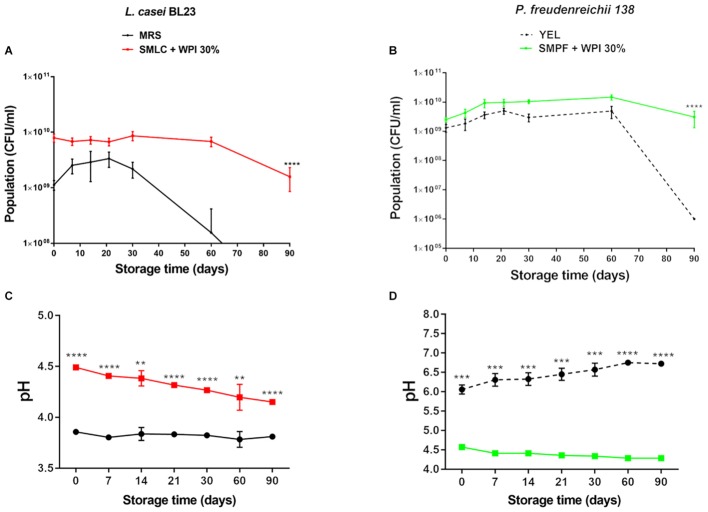
Viability of *L. casei BL23* and *P. freudenreichii* 138 are maintained during 90 days of cold storage when are cultured in skim milk supplemented with WPI. Final population (CFU ml^-1^) of **(A)** *L. casei* BL23, and **(B)** *P. freudenreichii* 138 in the indicated culture media. Bacteria were culture until the stationary phase and then storage at 4°C for 90 days. Viable bacteria were enumerated by counting the colonies on days 0, 7, 14, 21, 30, 60, and 90 after storage. **(C,D)** Variation of pH values throughout storage days. Asterisks represent statistically significant differences as follows: ^∗^*p* < 0.05, ^∗∗^*p* < 0.01, ^∗∗∗^*p* < 0.001, ^∗∗∗∗^*p* < 0.0001.

**FIGURE 4:**
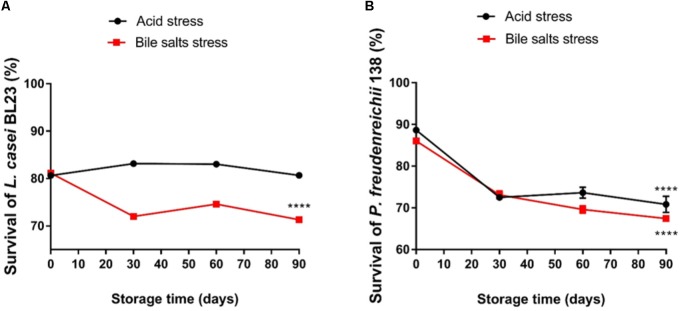
*Lactobacillus casei* BL23 **(A)** and *P. freudenreichii* 138 **(B)** maintain the ability to tolerate acid stress and bile salts stress even during storage when cultured in skim milk supplemented with WPI. Viable bacteria were enumerated by counting colonies on cultures after acid and bile salt stress on days 0, 30, 60, and 90 post-storage and then expressed as percent survival (means ± standard deviation). The assays were performed in triplicate technique and biological triplicate. Asterisks represent statistically significant differences as follows: ^∗^*p* < 0.05, ^∗∗^*p* < 0.01, ^∗∗∗^*p* < 0.001, ^∗∗∗∗^*p* < 0.0001.

Changes in bacterial population in fermented skim milk supplemented with whey protein were monitored during storage at 4°C for 90 days (**Figures 3A,B**). The viability of both strains, in skim milk contained 30% of WPI, remained practically unchanged, with a small CFU reduction after 90 days. After cold storage, the *L. casei* BL23 population in the skim milk culture media was maintained at 1.5 × 10^9^ CFU mL^-1^, while the *P. freudenreichii* 138 population at 7.4 × 10^9^ CFU mL^-1^. In contrast, viable *L. casei* BL23 and *P. freudenreichii* 138 was significantly decreased when grown in culture media (MRS and YEL, respectively) over the entire storage time, presenting a final population below 2 × 10^2^ CFU mL^-1^, for *L. casei* BL23 and 1.0 × 10^6^ CFU mL^-1^ for *P. freudenreichii* 138. **Figures 3C,D** represents the evolution of the pH values in the culture of both strains during the storage. We observed a decrease in pH values in beverages containing WPI. In the order hand, in controls the pH values did not decline over the storage time, remaining constant for *L. casei* BL23 while a slight increase in the pH value was detected for *P. freudenreichii* 138. **Figures 4A,B** represent the survival rates of *L. casei* BL23 and *P. freudenreichii* 138, upon acid and bile salt stresses, following storage at 4°C. The survival rate of *L. casei* remained almost constant between days 0 and 90 of storage (**Figure 4A**). For *P. freudenreichii* 138, the acid and bile salts tolerance slightly decreased, between days 0 and 30 (**Figure 4B**). However, propionibacteria survival remained around 70% after 90 days of storage.

### Probiotic Beverage Fermented by *L. casei* BL23 or *P. freudenreichii* 138 Reduces the Weight Loss in Mice With Mucositis

**FIGURE 5:**
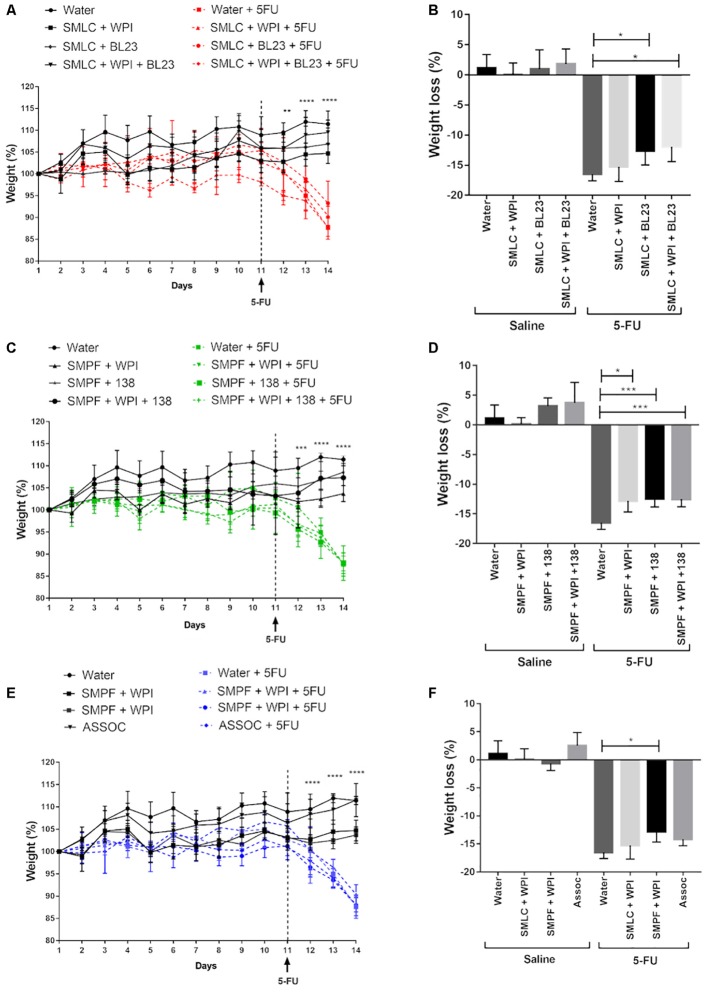
Time-course of body weight for mice treated with **(A)** probiotic beverage fermented by *L. casei* BL23; **(C)** probiotic beverage fermented by *P. freudenreichii* 138 and **(E)** probiotic beverage fermented by association with *L. casei* BL23 and *P. freudenreichii* 138. **(B,D,F)** Weight loss observed after 5-FU injection and differences across groups. *N* = 6–9. Mice were weighted daily during 14 days. Asterisks represent statistically significant differences as follows: ^∗^*p* < 0.05, ^∗∗^*p* < 0.01, ^∗∗∗^*p* < 0.001, ^∗∗∗∗^*p* < 0.0001.

Time-course of the weight of mice during the 14 experimental days is shown in **Figure 5**. None of the treatments with probiotic beverages was able to significantly alter the weight of mice during the first 11 days of gavage preceding mucositis induction. As expected, mice receiving 5-FU began to lose weight soon after the drug injection on day 11 (**Figures 5A,C,E**). Interestingly, we observed that the treatment with probiotic beverage fermented by *L. casei* BL23 (**Figure 5B**) or fermented by *P. freudenreichii* 138 (**Figure 5D**) was able to reduce the weight loss of inflamed mice, in the presence or absence of WPI in the medium. However, the association of both bacterial strains were not efficient to further limit the weight loss, compared to mice that received no probiotic treatment (**Figure 5F**).

### Beverage Supplemented With Whey Protein Isolate Improves Mucosal Preservation in the Inflamed Mice

**FIGURE 6:**
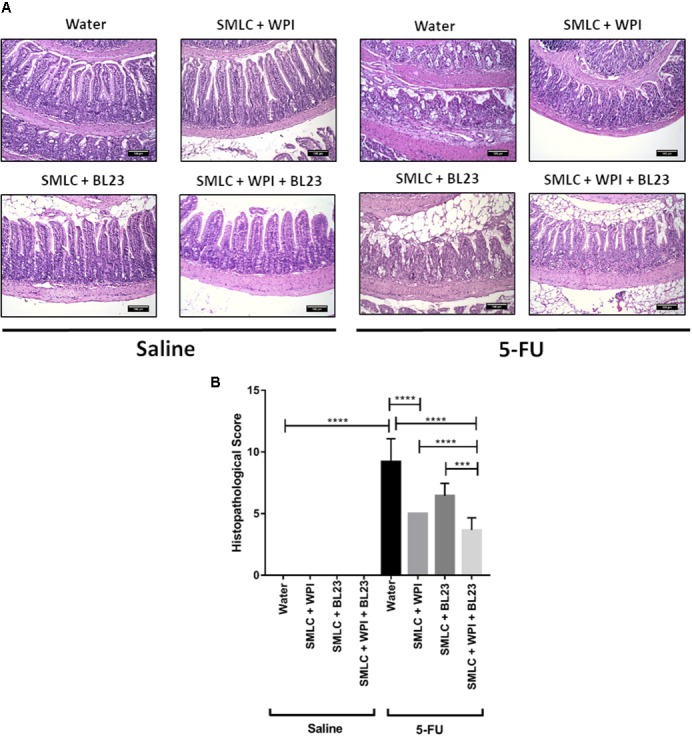
Administration of skim milk supplemented with WPI fermented by *L. casei* BL23 prevents mucosal damage in mice. **(A)** Representative H&E-stained images from mucosal histopathology and **(B)** histopathological score obtained in mice treated. The image acquisition was done with a 20× magnification objective. Scale bar = 100 μm. Same control groups were used for all experimental probiotic assays. Asterisks represent statistically significant differences as follows: ^∗^*p* < 0.05, ^∗∗^*p* < 0.01, ^∗∗∗^*p* < 0.001, ^∗∗∗∗^*p* < 0.0001.

**FIGURE 7:**
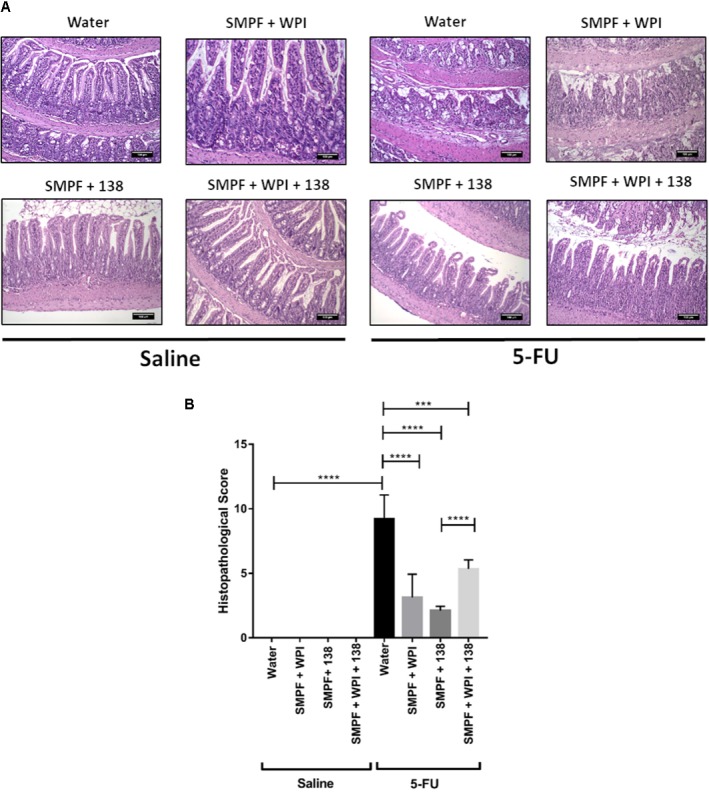
Administration of skim milk without supplemented with whey protein isolate and fermented by *P. freudenreichii* 138 prevents mucosal damage in mice. **(A)** Representative H&E-stained images from mucosal histopathology and **(B)** histopathological score obtained in mice. The image acquisition phase was done with a 20× magnification objective. Scale bar = 100 μm. Same control groups were used for all experimental probiotic assays. Asterisks represent statistically significant differences as follows: ^∗^*p* < 0.05, ^∗∗^*p* < 0.01, ^∗∗∗^*p* < 0.001, ^∗∗∗∗^*p* < 0.0001.

**FIGURE 8:**
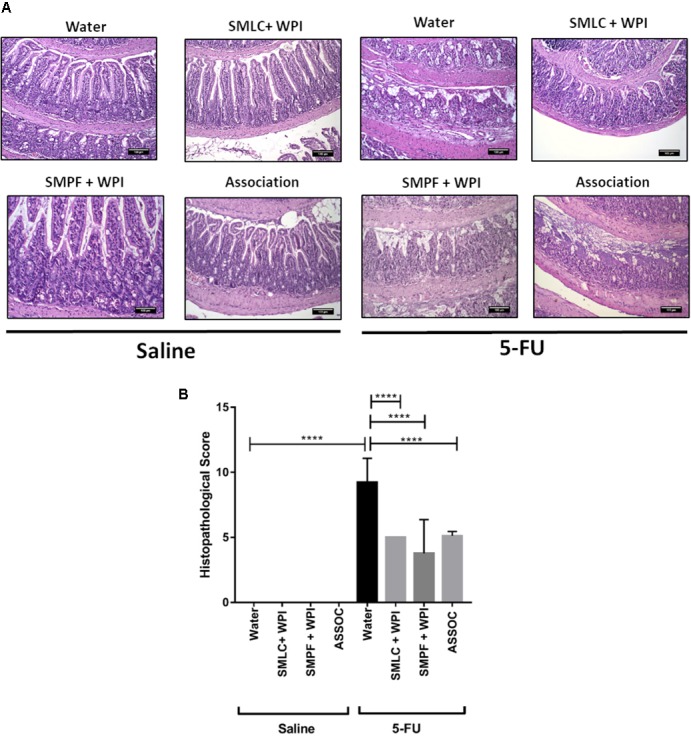
Association of skim milk fermented by *L. casei* BL23 and skim milk fermented by *P. freudenreichii* 138 does not provide additional effect in prevents mucosal damage. **(A)** Representative H&E-stained images from mucosal histopathology and **(B)** histopathological score obtained in the animals treated with different beverages. The image acquisition phase was done with a 20× magnification objective. Scale bar = 100 μm. Same control groups were used for all experimental probiotic assays. Asterisks represent statistically significant differences as follows: ^∗^*p* < 0.05, ^∗∗^*p* < 0.01, ^∗∗∗^*p* < 0.001, ^∗∗∗∗^*p* < 0.0001.

Histological analysis revealed a mucosal pattern within normal limits in all groups injected with saline (0.9% NaCl), showing that the probiotics beverages did not alter gut mucosal morphology (**Figures 6–8**). On the other hand, mice submitted to mucositis demonstrated alterations in the morphological structure of the ileum, which was evidenced by an increase in the histopathological parameters. This reflected mainly inflammatory cell infiltration in the *lamina propria*, submucosa and muscular layer, and a prominent alteration in villus structure. However, mice treated with probiotic beverages showed decreased mucosal damage, compared to inflamed mice that did not receive any probiotic treatment. Moreover, supplementation with WPI was able to improve the anti-inflammatory effects of *L. casei* BL23 beverage (**Figure 6**) but not for *P. freudenreichii* (**Figure 7**). Mice treated with the association of *L. casei* BL23 and *P. freudenreichii* 138 in skim milk, presented a reduced histopathological score compared to mice receiving only water, however, the histopathological scores obtained are no better than the treatment using the individually fermented milks by *L. casei* BL23 or *P. freudenreichii* 138 (**Figure 8**).

### Treatment With Probiotic Beverages Prevented Villus Shortening and Degeneration of Goblet Cells

**FIGURE 9:**
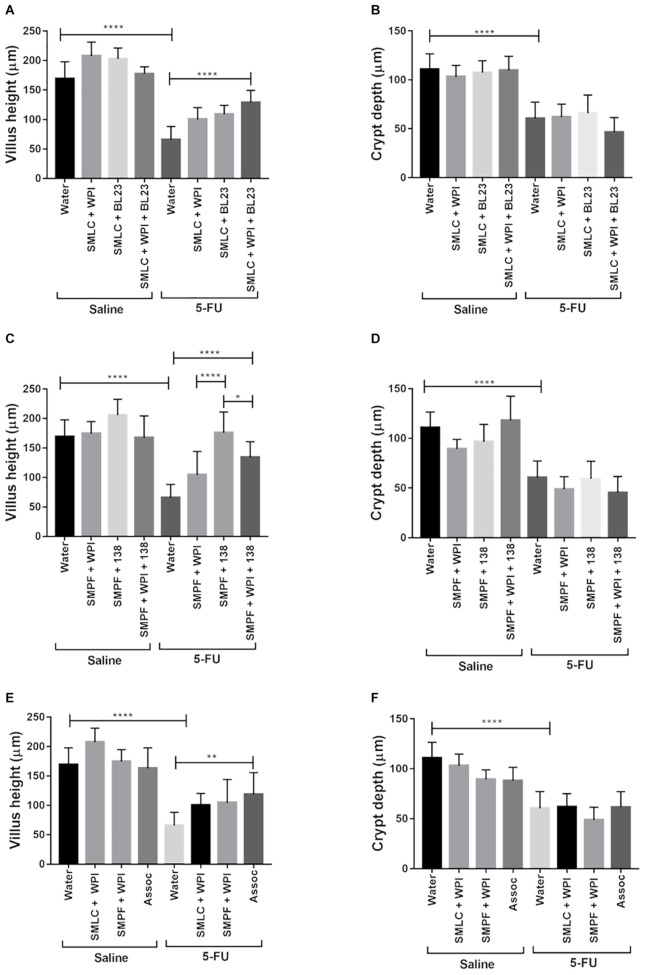
Administration of probiotic beverages improves villus architecture. Morphometric analysis of villus height and crypt depth of animals treated with **(A,B)** beverages fermented by *L. casei* BL23; **(C,D)** beverages fermented by *P. freudenreichii* 138 or **(E,F)** beverages fermented by the association of both bacteria following 5-FU or saline administration. Values were obtained by measuring ten random images of the ileum of mice. *N* = 6, 9. Asterisks represent statistically significant differences as follows: ^∗^*p* < 0.05, ^∗∗^*p* < 0.01, ^∗∗∗^*p* < 0.001, ^∗∗∗∗^*p* < 0.0001.

**FIGURE 10:**
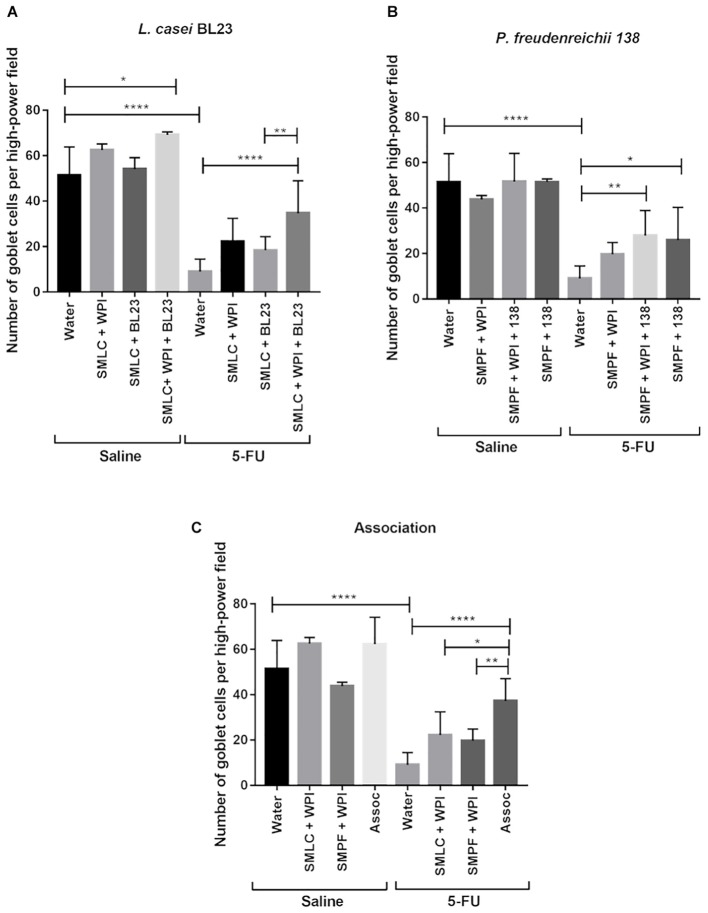
Administration of probiotic beverages prevented the marked degeneration of goblet cells in the mice ileum. Quantification of intact goblet cells in the animals ileum treated with **(A)** beverages fermented by *L. casei* BL23; **(B)** beverages fermented by *P. freudenreichii* 138 or **(C)** beverages fermented by the association of both bacteria following 5-FU or saline administration. Values were obtained by counting intact cells in ten random field images of mice. Results were expressed as means ± standard deviation. *N* = 6–9. Asterisks represent statistically significant differences as follows: ^∗^*p* < 0.05, ^∗∗^*p* < 0.01, ^∗∗∗^*p* < 0.001, ^∗∗∗∗^*p* < 0.0001.

Morphometric analysis was carried out to evaluate epithelial integrity. A decrease in villus height and in crypt depth was observed in mice after 5-FU injection (66 μm) (**Figure 9**). Treatment with probiotic beverages showed increased villus height, especially in groups treated with SMLC + WPI + BL23 (129.2 μm) (**Figure 9A**) and SMPF + 138 (176.04 μm) (**Figure 9C**). No difference was found in crypt depths either in treated or untreated mice. As expected, the mucositis induction resulted in substantial decrease in goblet cells number (9.08 goblet cell/hpf) (**Figure 10**) when compared to the groups injected with 0.9% saline (51.4 goblet cell/hpf). In the other hand, administration of probiotics beverages prevented the degeneration of goblet cells in the mice ileum. The highest goblet cell count was found in mice treated with SMLC + WPI + BL23 (34.7 goblet cell/hpf) (**Figure 10A**) and SMPF + 138 (27.9 goblet cell/hpf) (**Figure 10B**).

### Administration of Probiotic Beverages Did Not Change the Secretory IgA Production

**FIGURE 11:**
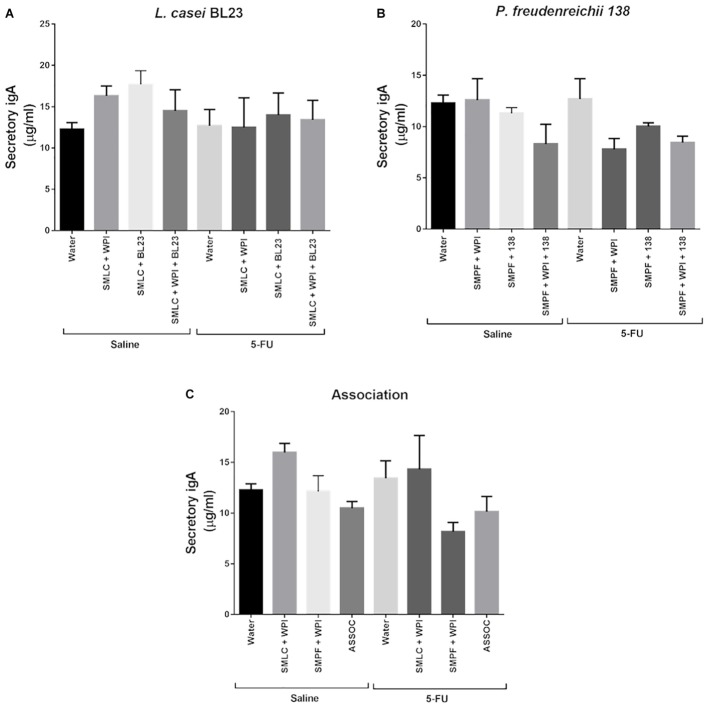
Administration of probiotic beverages did not alters the secretory IgA levels. Quantification of immunoglobulin A secretion (sIgA) in the small intestine of healthy or inflammation mice, treated for 14 days with **(A)** *L. casei* BL23 **(B)** *P. freudenreichii* 138, or **(C)** association of *L. casei* BL23 and *P. freudenreichii* 138. Results were expressed as means ± standard deviation. *N* = 6–9.

**Figure 11** indicates the concentration of IgA secreted in the small intestine of healthy animals or after induction of 5-FU mucositis, treated or not with *L. casei* BL23, with *P. freudenreichii* 138, or with the association of the two strains. Our results showed that there was no significant difference across the groups evaluated in this study.

## Discussion

Mucositis is a gastrointestinal inflammation that affects the quality of life of patients undergoing malignancy treatments (Sonis, 2004). Currently, classical therapies available for the prevention and treatment of the disease are not very effective and therefore new options have been suggested, such as the use of probiotic bacteria (Carvalho et al., 2017a). Several of these probiotics required the addition of protective matrices, in order to confer a protection via an efficient delivery of probiotic bacteria to the GIT, and enhance the therapeutics effects in the disease context (Carmo et al., 2017). The present study investigated the effects of whey protein isolate as a protective matrix for two bacterial strains and study the probiotic potential of these fermented beverages in a mucositis mice model induced by 5-FU.

Our data show that the addition of whey protein isolate boosted the growth of *L. casei* BL23 and of *P. freudenreichii* 138. This result may be due to a larger amount of nutrients provided by milk constituents and by WPI in the beverages, including carbohydrate and nitrogen sources, as they are essential for the energy metabolism of both *L. casei* BL23 and *P. freudenreichii* 138 (Cousin et al., 2011; Pessione, 2012). This result corroborates previous studies showing that other bacteria experienced enhanced growth as a result of whey proteins addition (Almeida et al., 2009; Skryplonek and Jasińska, 2015; Huang et al., 2016b; Baruzzi et al., 2017).

Due the fact that the products to be considered as a probiotic must be in sufficient quantities of viable bacteria in your local of action (Cousin et al., 2012a; Rabah et al., 2017), we decided to investigate the efficiency of WPI as a protective matrix for bacteria. For this, we evaluated the survival rate of *L. casei* BL23 and of *P. freudenreichii* 138 strains in environments that simulated stomach pH (pH 2), in the presence of bile salts in GIT and at high temperature. Our results demonstrated that the tolerance of *L. casei* BL23 and of *P. freudenreichii* 138 to these stressing conditions was significantly higher in the presence of WPI. Therefore, beverages containing WPI also showed a high viable cell counts after these stresses. Thus, skim milk supplemented with WPI is an effective matrix for the two strains used in this work, considering acid, bile salts and high-temperature stresses. Similar results were described by Vargas et al. (2015), who demonstrated that the probiotic strains *Streptococcus thermophilus* and *L. bulgaricus* presented a higher tolerance to the same stress conditions in the presence of WPI in the culture medium (Vargas et al., 2015). Cousin et al. (2012b) have also shown that *Propionibacterium* strains survived better in acid and biliary stresses when included in a dairy matrix (Cousin et al., 2012b). In a study by Huang et al. (2016b) was also demonstrated that the *P. freudenreichii* CIRM-BIA 129 tolerates simulated GIT stresses when included in hyperconcentrated sweet whey (Huang et al., 2016b). Studies suggest that dairy proteins protect probiotic bacteria via a process called coacervation. In this process, the proteins form microspheres that pack the microorganism inside, thus forming a kind of barrier that protects them from adverse environmental conditions (Silva et al., 2015; Coghetto et al., 2016).

The current definition of probiotic stipulates that microorganisms should be consumed alive (WHO, 2002; Hill et al., 2014). Thus, the survival of bacteria at low temperatures is an important parameter for the development of an effective probiotic product and fermented dairy products are generally stored at 4°C (Cousin et al., 2012b). Both bacteria tested in this work remained viable during at least 90 days at 4°C when cultured in skim milk supplemented with WPI, reaching up to 10^9^ CFU mL^-1^, after 90 days. Furthermore, it is possible to suggest that these bacteria were also metabolically active as a pH decrease was observed, due to production of lactic acid and propionic acid (main fermentation products of *L. casei* and *P. freudenreichii*, respectively) during cold storage. In addition, stress tolerance of *L. casei* BL23 and of *P. freudenreichii* 138 was maintained upon cold storage in skim milk plus WPI. Therefore, the amount of nutrients provided by milk plus WPI was sufficient to sustain survival of the bacteria over 90 days of cold storage, in accordance with previous reports (Cousin et al., 2012b; Vargas et al., 2015; Moslemi et al., 2016; Shori, 2016; Baruzzi et al., 2017).

In our probiotic beverages, WPI supplementation increases the bacteria’s survival rate to environmental stresses, which is an essential parameter for therapeutic effects (Rabah et al., 2017). Since, *L. casei* BL23 is able to reduce inflammation parameters in colitis model (Rochat et al., 2007; Watterlot et al., 2010), and *P. freudenreichii* 138 has been shown some probiotic effects *in vitro* and *ex vivo* model (Cousin et al., 2012a,b), which is interesting to check their potentials in other disease models. In this context, we tested whether the probiotic beverages were able to exert beneficial effects in mice submitted to experimental mucositis and whether the addition of WPI would enhance these probiotic effects. 5-FU treatment caused weight loss, shortening of intestinal villi and an inflammation of mucosa in mice, in accordance with the literature (Carvalho et al., 2017a). Moreover, probiotic beverages fermented by *L. casei* BL23 and *P. freudenreichii* 138 were able to decrease 5-FU-induced intestinal inflammation in BALB/c mice, with preservation of the mucosal integrity and reduced weight loss. Same results were observed by oral administration of Simbioflora^®^, that containing *L. paracasei, L. rhamnosus, L. acidophilus* and *Bifidobacterium lactis* plus fructooligosaccharide in a 5-FU-mucositis mice model and in a treatment using a probiotic mixture, named VSL#3, in mucositis model induced by Irinotecan in rats (Bowen et al., 2007; Trindade et al., 2018). Our study also shows that addition of WPI improved *L. casei* BL23 beneficial effects in the ileum, but not for *P. freudenreichii* 138.

Another important feature evaluated in this study was the number of goblet cells throughout the tissue. Goblet cells are responsible for producing a layer of mucus that covers the entire surface of the intestinal epithelium and is mainly composed of high molecular weight glycoproteins known as mucins (Johansson et al., 2013). This mucus prevents the direct adhesion of microorganisms to the epithelium and their translocation to the internal layers of the intestine, besides being important for the lubrication of the intestinal walls and for the protection of the epithelium against digestive acidic fluids and toxins (Kim and Khan, 2013). Previous studies have described that the intestine cells need a series of amino acids, mainly threonine, cysteine, and serine, for the synthesis of this mucus in healthy conditions (Faure et al., 2006). However, during inflammatory processes such as mucositis, a superactivation of the goblet cells occurs, aiming to increase the protection of the epithelium damaged by the inflammatory process (Stringer et al., 2007, 2009a). Consequently, the requirement for amino acids by the cells is increased. However, these amino acids are usually insufficient during the inflammation, compromising adequate mucus barrier functioning (Stringer et al., 2009b). The demand for threonine, cysteine, and serine can be adequately supplied by the diet in order to increase the availability of these amino acids (Faure et al., 2006). WPI used in this study is rich in these three amino acids. Our probiotic beverages prevented the degeneration of goblet cells, suggesting that the presence of milk and WPI may have increased the availability of these amino acids, increasing the production of mucus and consequently improving the framework of protection and tissue repair observed in the histological analyses. Similar results were shown in a probiotic treatment with *Saccharomyces cerevisiae* UFMG A-905 in a murine model of irinotecan-induced mucositis (Bastos et al., 2016), as well as in a mice treated with a mixture of *L. acidophilus* and *Bifidobacterium bifidum* in a 5-FU-induced intestinal mucositis model (Yeung et al., 2015). Prisciandaro et al. (2011) also shown that a *Escherichia coli* Nissle 1917 (EcN) probiotic derived supernatants was able to partially maintained acidic-mucin producing goblet cells in the jejunum and neutral mucin producing goblet cells in the ileum, in 5-FU mucositis model in mice (Prisciandaro et al., 2011). Furthermore, due to the capacity to preserve globet cells and consequently to maintenance of mucin production, this is possible that adhesion of *L. casei* BL23 and *P. freudenreichii* 138 strains to the intestinal epithelial cells can be enhanced in mouse GIT, leading to an increase the probiotic therapeutic effect (Ouwehand and Salminen, 2003).

In summary, our results indicate that the developed probiotic beverages have anti-inflammatory effects in mucositis. Thus, we sought to investigate the role of IgA in the regulation of inflammatory conditions in these mice. IgA is the main antibody type found in mucosal secretions (Pabst et al., 2016) and has many important functions such as modulation of intestinal microbiota and mucosal protection against invading pathogens (Lycke and Bemark, 2017). These functions are naturally important in intestinal mucositis because this disease is associated with dysfunctions related to imbalances in the intestinal microbial community (Clemente et al., 2012). Destruction of the physical barrier that covers the GIT facilitates the invasion of pathogens from the lumen (Bischoff et al., 2014). However, none of the probiotic beverages used in this study was able to alter IgA production, ruling out a probiotic effect via stimulation of sIgA. Accordingly, administration of another probiotic species, *Lactococcus lactis* NCDO, in a DSS-induced colitis model, does not enhance the levels of sIgA (Luerce et al., 2014).

## Conclusion

We have demonstrated that the supplementation of skim milk with 30% of whey protein isolate is a good matrix to provide protection for the *L. casei* BL23 and for *P. freudenreichii* 138 against environmental stresses. Furthermore, both probiotic beverages developed here were efficient in preventing mucositis induced by 5-Fluorouracil in BALB/c mice. *L. casei* BL23 protective effect was further enhanced by the addition of WPI. The benefits of adding WPI for the prevention of mucositis thus depends on the bacterial strain used.

## Author Contributions

BC performed the *in vitro* analysis, animal experimentation regarding mucositis pre-treatment with the probiotic strains, interpreted the data regarding the immunological parameters that were assessed, and was a major contributor in the writing of the manuscript. EO and BS were major contributors in the animal experimentation. EF and SS performed, analyzed, and interpreted the histological analysis from ileum slides. JA analyzed and interpreted the morphometric analysis. LA performed the *in vitro* analysis and data interpretation. LL and HA performed, analyzed, and interpreted the secretory IgA quantification assay. AF, AV, LG, and VA contributed to the data interpretation and were major contributors in the writing of the manuscript. GJ and YLL were responsible for ceding the strains, contributed to the data interpretation, and were major contributors in the writing of the manuscript. RC and FC have contributed equally in the supervision, performing experiments, analysis and interpretation of the immunological data, and were major contributors equally in the writing of the manuscript.

## Conflict of Interest Statement

The authors declare that the research was conducted in the absence of any commercial or financial relationships that could be construed as a potential conflict of interest.
